# The Influence of the Toxin/Antitoxin *mazEF* on Growth and Survival of *Listeria monocytogenes* under Stress

**DOI:** 10.3390/toxins9010031

**Published:** 2017-01-13

**Authors:** Thomas D. Curtis, Ippei Takeuchi, Lone Gram, Gitte M. Knudsen

**Affiliations:** Department of Biotechnology and Biomedicine, Technical University of Denmark, Matematiktorvet Bldg. 301, DK-2800 Kongens Lyngby, Denmark; thocu@bio.dtu.dk (T.D.C.); takenoko.no.satousan@gmail.com (I.T.); gmkn@bio.dtu.dk (G.M.K.)

**Keywords:** *Listeria monocytogenes*, sigma B (σ^B^), toxin-antitoxin modules, MazEF, antibiotics, sub-MIC, stress response, persister cells

## Abstract

A major factor in the resilience of *Listeria monocytogenes* is the alternative sigma factor B (σ^B^). Type II Toxin/Antitoxin (TA) systems are also known to have a role in the bacterial stress response upon activation via the ClpP or Lon proteases. Directly upstream of the σ^B^ operon in *L. monocytogenes* is the TA system *mazEF*, which can cleave mRNA at UACMU sites. In this study, we showed that the *mazEF* TA locus does not affect the level of persister formation during treatment with antibiotics in lethal doses, but exerts different effects according to the sub-inhibitory stress added. Growth of a Δ*mazEF* mutant was enhanced relative to the wildtype in the presence of sub-inhibitory norfloxacin and at 42 °C, but was decreased when challenged with ampicillin and gentamicin. In contrast to studies in *Staphylococcus aureus*, we found that the *mazEF* locus did not affect transcription of genes within the σ^B^ operon, but MazEF effected the expression of the σ^B^-dependent genes *opuCA* and *lmo0880*, with a 0.22 and 0.05 fold change, respectively, compared to the wildtype under sub-inhibitory norfloxacin conditions. How exactly this system operates remains an open question, however, our data indicates it is not analogous to the system of *S. aureus*, suggesting a novel mode of action for MazEF in *L. monocytogenes.*

## 1. Introduction

*Listeria monocytogenes* is a foodborne pathogenic bacterium, capable of causing major pregnancy complications or severe symptoms, such as septicaemia or meningitis, in immunocompromised persons with upwards of 30% mortality [1]. In addition to the inhospitable conditions of the human digestive system, *L. monocytogenes* has evolved to persevere in a several environments; from soil to the intentionally harsh conditions of food processing facilities, where it must cope with exposure to preservatives and disinfectants [2]. One of the most important factors in the resilience of this organism is the alternative sigma factor B (σ^B^), which under specific conditions directs RNA polymerase to transcribe >150 stress responsive genes [3,4] that allow this bacteria to cope with and even grow in NaCl concentrations as high as 6%, temperatures near 0 °C, and pH as low as 4.3 [5]. 

Several other systems are involved in bacterial survival during stress conditions, including the chromosomally encoded Toxin/Antitoxin (TA) systems, which are most widely known for their role in the formation of persister cells [6]. TA systems are thought to become stochastically activated in a small fraction of a bacterial population, leading to this reversible, dormant-like cell state that renders the bacterial cell invulnerable to killing by most classes of antibiotics [6]. One of the most widely studied TA systems is the type II TA MazEF*.* Like all Type II TA systems, the *mazEF* genes are co-transcribed into two proteins, MazE, an unstable antitoxin that binds and inhibits activity of the toxin MazF, an endoribonuclease that cleaves mRNA at specific sites. Under activating conditions of stress in *Escherichia coli*, *mazEF* transcription is reduced leading to the degradation of MazE by the ClpP or Lon proteases [7,8], thereby freeing up the toxin to induce stasis via the cleavage of mRNA at ACA sites [9]. In *Staphylococcus aureus*, which lacks the Lon protease, MazE is degraded only by the ClpP protease [8] and MazF mRNA cleavage is more specific, occurring at UACAU sites [10]. Persister cells have previously been observed in *L. monocytogenes*, where the authors speculated that they may result from TA systems [11]. *L. monocytogenes* EDGe has two predicted type II TA systems in its chromosome [12], which is relatively few for free-living prokaryotes [13]. The first is an experimentally verified *mazEF* TA system that has been shown to induce dormancy and cleave mRNA at UACMU sites upon overexpression [14], while the second is a putative TA system belonging to the Xre-COG2856 family.

In addition to persister formation, an increasing body of evidence suggests that TA modules also have a wider regulatory role in stress-related processes, such as the response to nutrient starvation [15] and biofilm formation [16]. Interestingly, the *mazEF* TA module has been found to be located directly downstream of the stringent response regulator *relA* in Gram negative bacteria like *E. coli* [17] and directly upstream of σ^B^ in Gram positive bacteria such as *Staphylococcus aureus* [18], *Bacillus subtillis* [19]**,** and *L. monocytogenes*. Reasons for this synteny have so far been elusive, however, Donegan and Cheung [18] have shown that σ^B^ of *S. aureus* auto-regulates the σ^B^ operon through the *mazEF* promoter in a stress specific manner. MazF has also been shown to influence transcript levels of genes within the σ^B^ operon [20,21], indicating that it may act as another level of regulation in the σ^B^ response.

A second proposed mechanism of action for MazEF presumes that specific genes have evolved to contain unusually high abundances of the MazF cleavage site making them more susceptible to degradation by the toxin under conditions where MazE is degraded. One example is the gene *sraP* of *S. aureus*, which encodes the pathogenic adhesive factor SraP and contains 43 MazF cleavage sites compared to the 11 expected by chance [10]. Furthermore, when the top MazF cut-site rich genes of *S. aureus* are grouped according to biological function, the pathogenic factor gene group is significantly over-represented [10], whereas the putative MazF target genes in *Bacillus subtilis* [22] and *Staphylococcus equorum* [23] are mostly involved in the production of secondary metabolites or metabolism, respectively, suggesting that MazF may regulate different specific cellular processes depending on the bacteria.

Transcriptomic data from other studies in *L. monocytogenes* [24,25] show that *mazEF* (*lmo0887-0888*), one of only two type II TA systems predicted in the genome of *L. monocytogenes* EDGe [12], is also co-transcribed in the σ^B^ operon. Thus, given the role of the *mazEF* loci in other organisms, its proximity to the major stress response regulator σ^B^, and the finding that *L. monocytogenes* forms persister cells via an unknown mechanism [11], our purpose in this study was to investigate the potential role of this toxin/antitoxin system in the stress response of *L. monocytogenes.*

## 2. Results

### 2.1. Deletion of the mazEF TA System does not Detectably Alter Persister Formation

**Figure 1:**
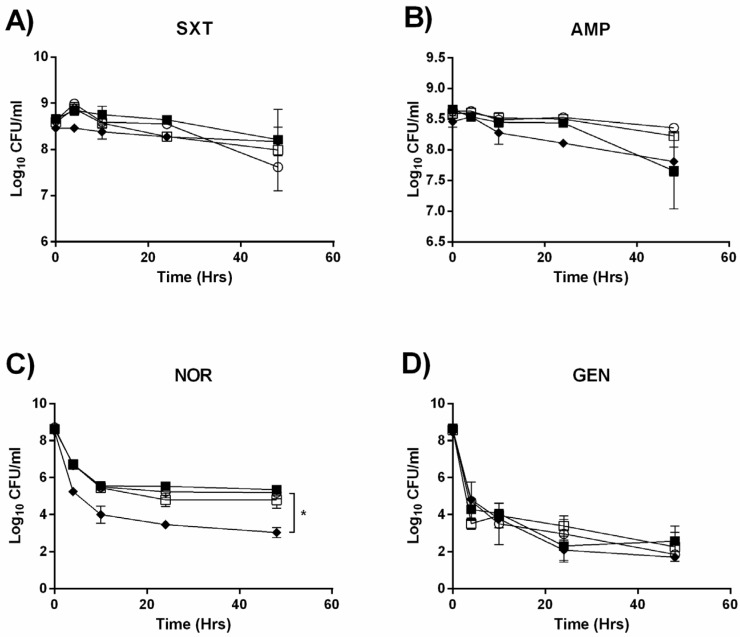
Killing kinetics of EDGe (○), Δ*sigB* (■), Δ*clpP* (♦), and Δ*mazEF* (□) exposed to approximately 30× Minimum Inhibitory Concentrations (MIC) for 72 h under shaking at 250 rpm with (**A**) co-trimoxazole (10 µg/mL); (**B**) ampicillin (3 µg/mL); (**C**) norfloxacin (100 µg/mL); and (**D**) gentamicin (10 µg/mL). Error bars represent standard deviation of the mean for three biological replicates. * *p* < 0.001.

Due to an abundance of evidence showing the direct role of TA systems in the formation of persister cells [26,27,28], we initially tested whether or not the deletion of one out of the two predicted TA systems in *L. monocytogenes* EDGe would negatively impact survival when challenged with high concentrations of antibiotics. Upon treatment with co-trimoxazole and ampicillin (Figure 1A,B), bacterial counts of each strain did not significantly differ and remained stable throughout the 48 h of exposure. All strains treated with norfloxacin exhibited a biphasic killing curve (Figure 1C), emblematic of persister cells with an initial steep decline in CFU/mL over the first 10 h, followed by a stationary plateau between 4 and 3 Log_10_ CFU/mL for the Δ*clpP* mutant and between 5.5 and 5.7 Log_10_ CFU/mL for the rest. Treatment with gentamicin resulted in a similar biphasic killing curve for all strains (Figure 1D). The only significant difference observed in CFU/mL to EDGe by the end of each 48 h treatment period was the Δ*clpP* mutant exposed to norfloxacin (*p* = 0.001).

### 2.2. MazEF Affects Growth under Specific Sub-Inhibitory Growth Conditions

**Figure 2:**
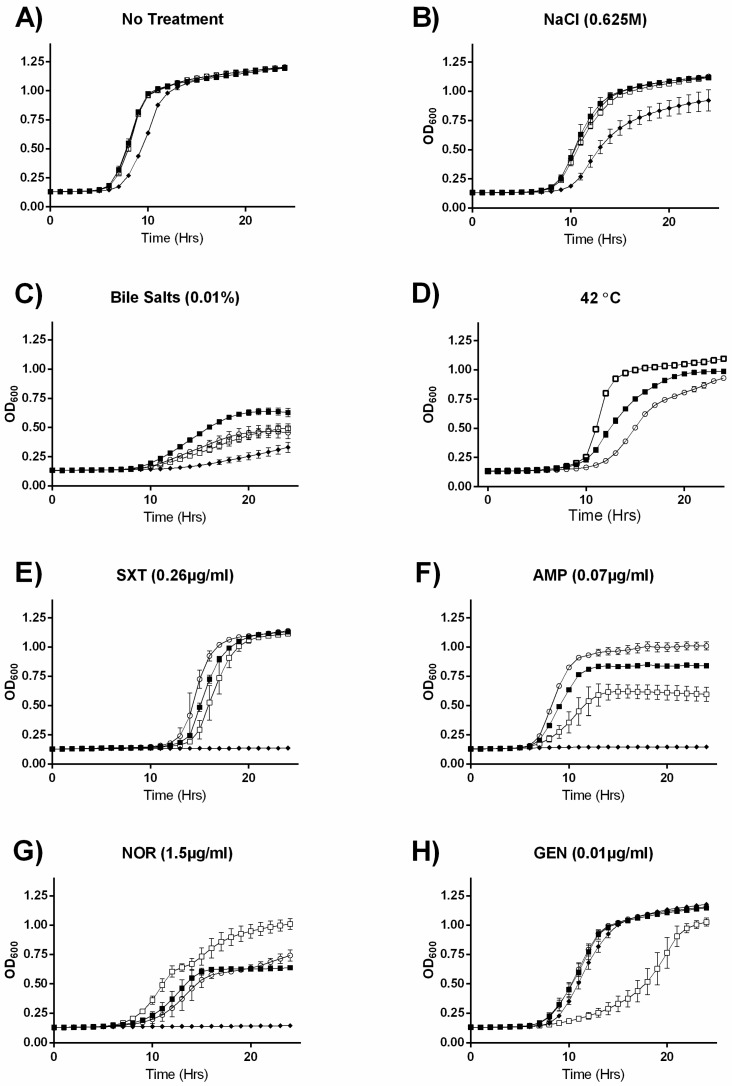
Growth curves for EDGe (○), Δ*sigB* (■), Δ*clpP* (♦), and Δ*mazEF* (□) exposed to sub-inhibitory stress. OD600 was measured in 96-well plates over 24 h for strains receiving (**A**) no treatment; (**B**) 0.625 M NaCl; (**C**) 0.01% bile salts; (**D**) 42 °C; (**E**) 0.25 µg/mL co-trimoxazole (SXT); (**F**) 0.07 µg/mL ampicillin (AMP); (**G**) 1.5 µg/mL norfloxacin (NOR); and (**H**) 0.10 µg/mL gentamicin (GEN). Error bars represent standard deviation of the mean for two biological replicates.

Since no link between the *mazEF* TA system and persister formation was observed, we hypothesized that it could have an active regulatory function, in which case it may require time to initiate in response to a stress. Therefore, we investigated the effects of the gene knockouts on growth under sub-inhibitory stress conditions: specifically, we selected clinically relevant antibiotics, as well as conditions known to be alleviated by the σ^B^ response. When grown in the absence of treatment (Figure 2A) all strains grew identically, with the exception of the Δ*clpP*, which was impaired in the exponential growth phase. Relative growth trends in NaCl and bile salts (Figure 2B,C) were essentially identical to growth in Brain Heart Infusion (BHI) alone, with the exception that the Δ*sigB* mutant had a growth advantage when grown in the presence of bile salts. When grown at 42 °C, the Δ*mazEF* strain had a stark growth advantage over the EDGe wildtype strain, and the Δ*sigB* mutant displayed a moderate growth advantage (Figure 2D). In contrast to the killing kinetic experiment, we observed significant differences in growth between the mutants and EDGe when grown in the presence of sub-inhibitory concentrations of antibiotics (Figure 2E–H). The Δ*clpP* mutant was more sensitive to all but gentamicin, where its relative growth deficiency to the other strains was abolished (Figure 2H). For the Δ*sigB* mutant, we observed slight growth disadvantages relative to EDGe under co-trimoxazole (Figure 2E) and ampicillin (Figure 2F) stress. The Δ*mazEF* mutant grew more poorly than EDGe in the presence of co-trimoxazole (Figure 2E) and ampicillin (Figure 2F) and was severely inhibited in gentamicin (Figure 2H). However, similar to growth at 42 °C, Δ*mazEF* grew faster and to a higher cell density when challenged with norfloxacin (Figure 2G).

In summary, all strains grew identically (except for the Δ*clpP* mutant) in the absence of stress, however, we observed unique growth differences between the strains depending on the stress added. We observed similarities between the Δ*sigB* and Δ*mazEF* mutants, which grew better at 42 °C and worse in the presence of ampicillin, with the Δ*mazEF* exhibiting a more extreme difference relative to the wildtype in the two conditions, as well as some growth patterns specific for the Δ*mazEF* mutant, which grew better than the rest of the strains in the presence of norfloxacin, but worse in gentamicin.

### 2.3. MazF Does not Detectably Degrade Putative Target Transcripts In Vivo

Given the established in vitro function of MazF in *L. monocytogenes* as an endoribonuclease that cleaves mRNAs at UACMU motifs [14], we compared the actual number of MazF cleavage sites in each gene of *L. monocytogenes* EDGe to the predicted number of MazF cleavage sites, as determined by gene length and nucleotide content. This was done based on the assumption that genes which have a much higher abundance of MazF cleavage sites, as compared to chance alone, will be more prone to the ribonucleic activity of MazF. Table 1 shows the 10 genes with the highest frequency of MazF cleavage sites, which ranged from functions involved in the electron transport chain (*lmo2638*), cell wall biogenesis and modification (*lmo1090* and *lmo0835*, respectively), and sugar uptake (*bvrB*) and metabolism (*pgi*).

**Figure 3:**
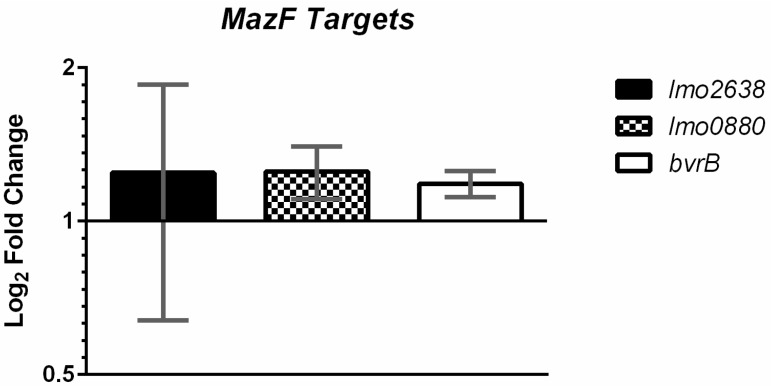
Quantification of putative MazF target mRNA via RT-qPCR. Fold-change in Log_2_ scale of the Δ*mazEF* mutant relative to EDGe under non-stressed conditions is shown for *lmo2638*, *lmo0880*, and *bvrB*, with 1, 2, and 0 MazF cleavage motifs within the amplicon, respectively. Error bars represent standard deviation of the mean for two biological replicates.

**Figure 4:**
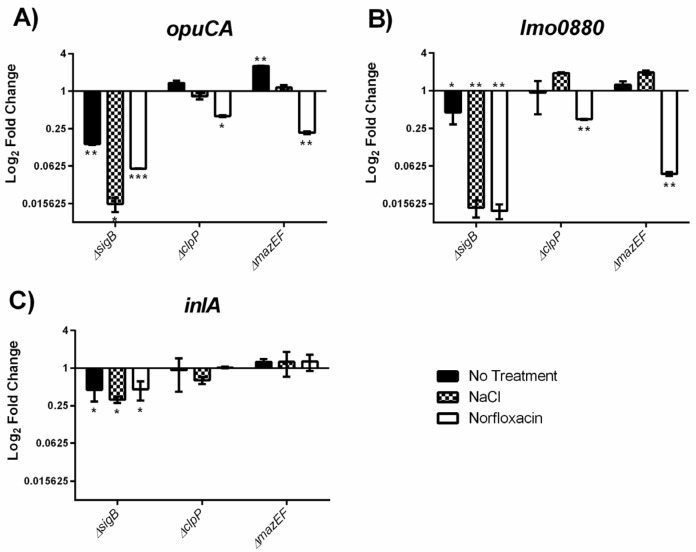
Quantification of σ^B^-dependent gene expression via RT-qPCR. Fold-change in Log_2_ scale of each gene relative to EDGe is shown for the (**A**) *opuCA*; (**B**) *lmo0880*; and (**C**) *inlA* genes*.* Each strain was grown at 37 °C with 200 rpm shaking and received either no treatment (MQ), osmotic stress (0.5 M NaCl), or sub-MIC (1 µg/mL) norfloxacin. *C*t values were normalized to the 16S rRNA gene (Δ*C*t), and then compared to EDGe expression (ΔΔ*C*t), and the average was taken from two independent experiments. Significance was determined using a *t*-test on the log_2_ transformed 2**^−^**^ΔΔ*C*t^ values. * *p* < 0.05, ** *p* < 0.0005, *** *p* < 0.000005.

To test if we could observe MazF mediated mRNA cleavage in vivo, we then measured the transcription of three genes with the high frequencies of MazF cut sites: *bvrB*, *lmo2638*, and *lmo0880* in EDGe and the Δ*mazEF* mutant under non-stressed conditions. RT-qPCR showed a slight, but non-significant increase in the number of the three transcripts present in Δ*mazEF*, as compared to EDGe (Figure 3). We also measured the expression of *lmo2638* in *L. monocytogenes* grown under sub-inhibitory concentrations of NaCl and norfloxacin, but similarly observed no significant difference between Δ*mazEF* and EDGe (data not shown). The mRNA level of *lmo0880*, a σ^B^ dependent gene, was also measured under sub-inhibitory NaCl and norfloxacin stress (Figure 4B).

### 2.4. MazEF Affects Expression of sigB Dependent Genes, but not sigB Operon

In order to test if the observed differences in growth were due to a MazEF mediated alteration of the σ^B^ response, we measured the expression of the σ^B^ dependent genes *opuCA* (glycine betaine-carnitine-choline ABC transporter ATP-binding protein), *lmo0880* (wall associated protein precursor LPXTG motif), and *inlA* (internalin A) in our strains grown without treatment, under osmotic stress (0.5 M NaCl), or with a sub-MIC (Minimum Inhibitory Concentration) (1 µg/mL) of norfloxacin. As expected, expression of each σ^B^ dependent gene (Figure 4) was decreased in the Δ*sigB* mutant in each of the tested conditions with the greatest reductions observed for *opuCA* and *lmo0880* under stress treatment. Interestingly, in the Δ*mazEF* mutant, we observed a treatment specific response in *opuCA* and *lmo0880* expression relative to EDGe. We observed no effect on *opuCA* expression in Δ*mazEF* cultures treated with NaCl, however, in untreated Δ*mazEF* cultures, *opuCA* expression increased when compared to the wildtype (2.549 fold change; *p* = 0.00006) and decreased when exposed to sub-MIC norfloxacin (0.215 fold change; *p* = 0.0004). This trend was reflected for *opuCA* expression in the Δ*clpP* mutant, although the only significant difference (*p* = 0.0008) was a 0.400 fold change when treated with norfloxacin (Figure 4A). We also observed a significant reduction (0.047 fold change; *p* = 0.00001) of *lmo0880* expression in the Δ*mazEF* strain under norfloxacin stress, but no significant difference under no treatment or NaCl treatment. These results were again reflected in the Δ*clpP* mutant, where *lmo0880* expression decreased under norfloxacin pressure (0.348 fold change; *p* = 0.00005) and demonstrated similar but non-significant trends in the other two treatment groups (Figure 4B). Expression of *inlA* was not significantly different in the Δ*clpP* or Δ*mazEF* mutants (Figure 4C).

These results show a positive influence of σ^B^ on *opuCA*, *lmo0880*, and *inlA* expression under a range of conditions as excepted. On the other hand, the *mazEF* gene products appear to repress *opuCA* expression when cells are unstressed, but, along with ClpP, activate *opuCA* and *lmo0880* expression when exposed to norfloxacin.

**Figure 5:**
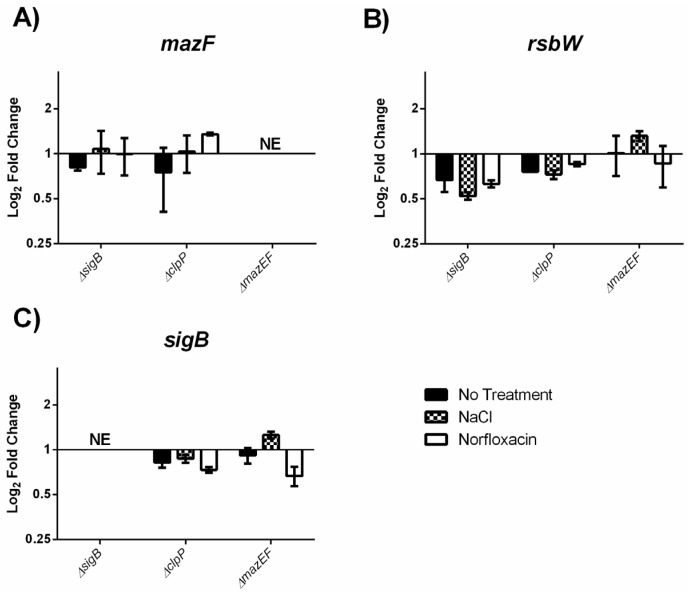
Quantification of σ^B^ operon expression via RT-qPCR. Fold-change in Log_2_ scale of each gene relative to EDGe is shown for the (**A**) *mazF*; (**B**) *rsbW*; and (**C**) *sigB* genes*.* Each strain was grown at 37 °C with 200 rpm shaking and received either no treatment (MQ), osmotic stress (0.5 M NaCl), or sub-MIC (1 µg/mL) norfloxacin. *C*t values were normalized to the 16S rRNA gene (Δ*C*t), and then compared to EDGe expression (ΔΔ*C*t), and the average was taken from two independent experiments. Significance was determined using a *t*-test on the log_2_ transformed 2**^−^**^ΔΔ*C*t^ values. NE: No Expression detected.

We investigated whether the observed MazEF mediated modification of the σ^B^ response was the result of transcriptomic regulation of *sigB* by *mazEF*, or vice-versa, by quantifying the gene expression of *mazF*, *rsbW* (anti-σ^B^ factor), and *sigB* in our strains grown under the same treatment conditions. The log_2_ adjusted fold change of *mazF* (Figure 5A), *rsbW* (Figure 5B), and *sigB* (Figure 5C) expression relative to EDGe was less than two-fold for all of the mutants, signifying that neither σ^B^, ClpP, or MazEF detectably influences the expression of these genes.

**Figure 6:**
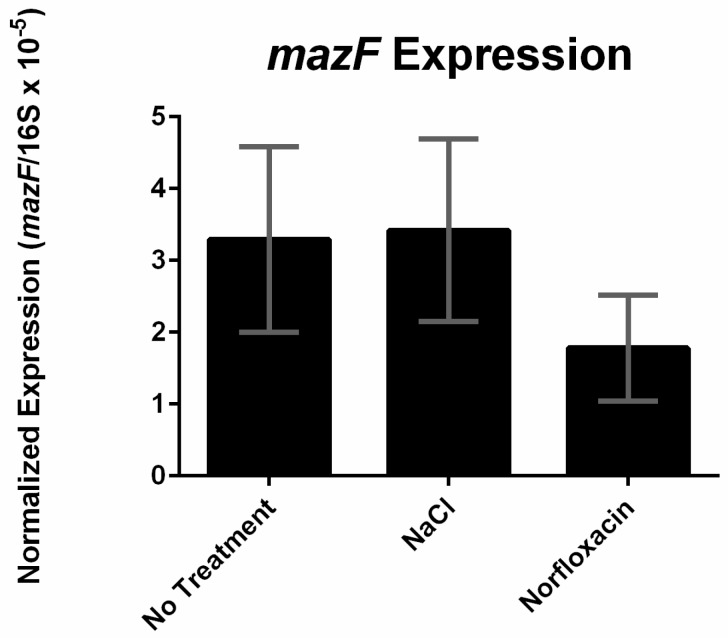
Expression of *mazF* under different stress conditions (no treatment, 0.5 M NaCl, or 1 µg/mL norfloxacin). The copy number of *mazF* transcripts were calculated and normalized to the copy number of 16S rRNA transcripts. Error bars represent standard deviation of the mean for two biological replicates.

As previously mentioned, the promoter of mazEF acts as a stress specific driver of sigB transcription in *S. aureus* [18]. Here we investigated if the mazEF promoter in *L. monocytogenes* is also stress responsive by measuring the gene expression of mazF in EDGe cultures receiving either no treatment or exposure to stress (osmotic shock and sub-MIC norfloxacin). We could not observe a significant difference in expression of mazF across the different treatment groups (Figure 6), signifying that the promoter of mazEF in *L. monocytogenes* is not responsive to the stress conditions tested.

## 3. Discussion

Given the established function of the *mazEF* toxin/antitoxin system in the stress response of several bacterial species [9,21,26,29,30], as well as its synteny to the major stress response regulator σ^B^, we hypothesized that this TA system could have an observable role in the response of *L. monocytogenes* to stress. As chromosomally encoded toxin/antitoxin systems are associated with persister cell formation and tolerance to antibiotics in other bacteria [6,31], we initially conjectured that the *mazEF* TA system in *L. monocytogenes* would serve a similar function, but this was not the case. We then hypothesized that the *mazEF* TA system could actively regulate the response of *L. monocytogenes* towards stress, which is supported by the observation that *mazEF* exerts an effect on the growth of this bacterium under specific sub-inhibitory growth conditions. TA systems have long been known to induce stasis [32,33], however, this is, to our knowledge, the first report of a more nuanced role, whereby the TA system exerts different effects according to the stress (i.e., enhanced growth of the Δ*mazEF* mutant relative to the wildtype in the presence of norfloxacin and 42 °C, but decreased growth when challenged with ampicillin and gentamicin) and agrees with the idea that the major function of most TA systems is to serve as a flexible response of a bacterial cell to stress conditions [28,34].

According to the Toxin/Antitoxin Database [12], type II TA systems are highly redundant in many bacterial chromosomes, such as *E. coli* MG1655 strain (16 predicted), *Salmonella* Typhimurium str. LT2 (18 predicted), and *Mycobacterium tuberculosis* H37Rv (77 predicted). This redundancy means that in some bacteria, multiple TA systems must be removed before an effect on persister formation can be observed [30,35,36]. Thus, it may be possible that the second predicted TA system of *L. monocytogenes* EGDe from the Xre-COG2865 family is acting redundantly, however, this TA family is essentially uncharacterized and to our knowledge has not been linked to the formation of persister cells. Furthermore, the MazEF TA system appears to exert a dominant effect in certain cases, in that a single deletion of this TA system can have significant effects on the formation of persister cells in *E. coli* [7] and *S. aureus* HG003 (nearly isogenic to NCTC8325), even when challenged with a bacteriostatic antibiotic [21]. Conversely, a recent study in *S. aureus* Newman found that knocking out all known TA systems (including *mazEF*) had no effect on the level of persisters when challenged with antibiotics, and that persister cells in this Gram positive bacteria are instead the result of a stochastic entry into the stationary phase, which the authors speculated could hold true for all Gram positive bacteria [37]. Fittingly, the prediction that *L. monocytogenes* only has two TA systems [12] and our observations that one of these had no detectable effect on the rate of persister formation is supportive of this theory.

*clpP* was the only gene in this study found to affect persister formation. Whether this is due to the bacteria being unable to degrade the antitoxin from the second predicted type II TA system of the Xre-COG2856 family (*lmo0113-0114*), or is the result of an already stressed cell due to an inability to clear toxic levels of altered proteins [38], is unknown. However, a reduction in persister cells has recently been shown in a Δ*clpP S. aureus* mutant, where the Δ*clpP* persister cells were more sensitive to an antibiotic from the β-lactam class (oxacillin), but not to a fluoroquinolone (levofloxacin) [39], whereas we observed the opposite with *L. monocytogenes*. The fact that β-lactams target cell wall biogenesis [40] and fluoroquinolones replicating DNA [41], suggests different roles for ClpP in the formation of persister cells between these two organisms.

The majority of studies on the MazEF TA system have relied on the ectopic overexpression of the toxin to identify its target genes and physiological effects. We, however, employed a strictly genetic approach, comparing only the presence or absence of the *mazEF* loci, as we feared that overexpression could have led to false positives not reflective of this TA system in its natural state. For instance, it seems to us that the conclusion that MazF induces dormancy in *S. aureus* [20] and *L. monocytogenes* [14] may be an artefact of the artificially high levels of toxin in the bacteria. While it appears to be true for *E. coli* [7], the observation that no changes in the level of persister cells (a proxy for dormancy) can be observed in ∆*mazEF* knockouts of *S. aureus* [37], and as we have shown here for *L. monocytogenes*, indicates that under natural circumstances the toxin does not accumulate in high enough concentrations in these Gram positive bacteria to induce dormancy. This reasoning was also partially why we did not perform a complementation experiment for the ∆*mazEF* knockout, as we feared that even a minor deviation from the natural levels of MazEF may introduce an artefactual result in the complemented strain. Additionally, we believe that the probability of an off-target mutation occurring in this study was sufficiently low to justify omitting a complementation experiment, given the method of allelic replacement we used, which employed two flanking regions with ~450 bp homology to target and delete each gene.

Due to the ribonuclease nature of the MazF toxin in vitro [14], as well as its location within the σ^B^ operon, we then hypothesized that mazEF could serve in more of an active regulatory role and that any MazF, or potentially related σ^B^ mediated effects would require time to manifest. If true, effects of the mazEF deletion would likely be undetectable when challenged with a sudden exposure to high concentrations of antibiotics. To test this, we measured the effect that each knockout had on growth in sub-inhibitory concentrations of different stressors. We first tested conditions known to stimulate a strong σ^B^ response in *L. monocytogenes*, specifically NaCl [42], bile salts [43]**,** and heat [44]. Interestingly, the only effect of MazEF on σ^B^ associated stress conditions was the enhanced growth of the Δ*mazEF* mutant at 42 °C, suggesting it plays a role in the thermo-tolerance of *L. monocytogenes* and is likely the result of heat induced upregulation of mazF [18,45] and/or the ClpP [46] protease, observed in other organisms. We also observed better growth of the Δ*mazEF* mutant when grown in sub-inhibitory concentrations of norfloxacin, signifying that under certain conditions, the toxin becomes active and slows the growth of *L. monocytogenes*, presumably in order to protect the bacteria by reducing the activity of antibiotic targets [47], which is consistent with studies showing that ectopic induction of artificially high levels of MazF induces stasis in other bacteria [20,48,49].

In contrast, when grown in sub-inhibitory concentrations of ampicillin, the Δ*mazEF* mutant grew more poorly than the wild type, which correlates with a study showing that Δ*mazEF* mutants of *S. aureus* also become more sensitive to β-lactams, where the authors speculate that it may be due to one or more unknown targets of MazF involved in cell wall synthesis or turnover [21]. We also observed an enhanced sensitivity of the Δ*mazEF* mutant towards gentamicin. This suggests MazEF exerts a protective effect by reducing the membrane potential of the bacteria, as aminoglycosides like gentamicin are dependent upon this for their uptake into cells [50]. Given that MazF in *L. monocytogenes* has been established in vitro as an endoribonuclease that cleaves mRNAs at UACMU motifs [14], as well as the demonstrated cleavage of the top putative MazF target genes in *S. aureus* upon *mazF* overexpression [10], it is tempting to explain these growth results through cleavage of the putative MazF target genes, such as those predicted to be involved in cell wall biogenesis and modification (*lmo1090* and *lmo0835*, respectively) and the electron transport chain (*lmo2638*, a NADH dehydrogenase). However, we were unable to detect a significant increase in the transcript levels of the three putative MazF target genes we tested in the ∆*mazEF* mutant as compared to the wildtype. The discrepancy between the MazF mediated mRNA cleavage in vitro and a lack of cleavage in vivo has also been observed in *S. aureus* and is thought to be the result of protective RNA binding proteins [20]. Furthermore, the observation that *lmo0880*, which is both a σ^B^ dependent and putative MazF target gene, is significantly decreased under norfloxacin stress in the ∆*mazEF* mutant contrasts with the idea of MazF mediated cleavage of transcripts containing high frequencies of the UACMU motif in *L. monocytogenes.*

We also investigated the possibility that MazEF affects growth under stress by modifying the σ^B^ response, which is supported by our observation that MazEF activates the expression of the σ^B^ dependent genes *opuCA* and *lmo0880* under norfloxacin stress. However, we observed that *inlA* expression was not affected by the absence of MazEF, but speculate that the effect may be diluted for this gene due to its co-regulation by the global virulence regulator PrfA [51]. In a recent study on the transcriptomic response to sub-lethal antibiotics [25], *L. monocytogenes* was found to significantly down-regulate the expression of certain σ^B^ dependent genes, including *opuCA* and *lmo0880*, in response to all tested antibiotics. Our observations suggest that MazEF tempers this antibiotic induced downregulation of these genes, thereby acting as a negative feedback mechanism to fine tune this response, which is consistent with the hypothesis that TA systems act as a quality control mechanism for gene expression under stress [28].

In an attempt to characterize the direct mechanism through which MazEF influences the expression of *opuCA*, *lmo0880*, and potentially other σ^B^-dependent genes, we first examined whether or not MazEF directly modifies the expression of genes within the σ^B^ operon, as has been observed in *S. aureus* for *sigB* [20] and *rsbW* [21], however, we were unable to observe any effect on transcript levels of either gene in the presence or absence of *mazEF*. We then tested another link observed in *S. aureus* between *mazEF* and the σ^B^ operon discovered by Donegan and Cheung [18] who, using an experimental setup similar to ours, showed that transcripts of *mazF* in *S. aureus* were significantly elevated in their Δ*sigB* strain, and that the complete 3.7 kb σ^B^ operon transcript (*mazEF*-*sigB*) was present only when exposed to certain conditions, such as heat shock or sub-inhibitory concentrations of erythromycin and tetracycline, but not to vancomycin. This, in effect, means that the promoter of *mazEF* in *S. aureus* acts as a stress specific promoter for the σ^B^ operon that is auto-repressed by σ^B^, thereby allowing the bacterium to fine tune its response to particular stimuli. This would explain the conditional effect of *mazEF* on *opuCA* and *lmo0880* expression, however, we observed no significant effects of either sub-inhibitory NaCl or norfloxacin on the expression of *mazF*, which would be expected if the promoter of *mazEF* in *L. monocytogenes* were acting in an analogous manner. Furthermore, σ^B^ had no effect on the expression of *mazF* in our study, or *mazE* as reported by other transcriptomic studies on Δ*sigB* mutants of *L. monocytogenes* [52,53], indicating that it does not act in the auto-regulatory manner observed in *S. aureus*. Our results suggest that *mazEF* modulates the σ^B^ response via some novel mechanism in *L. monocytogenes*, perhaps by cleaving the mRNA of the protein or proteins responsible for affecting the antibiotic induced downregulation of these genes.

## 4. Conclusions

At the physiological level, *mazEF* appears not to stop growth, as evidenced by its null effect on dormant persister cell formation, but rather modulate growth in response to specific stress factors. At the transcriptional level, MazEF modifies the expression of certain σ^B^ dependent genes like *opuCA* and *lmo0880* in a stress specific manner*.* Interestingly, with the exception of heat, *mazEF* seems immaterial with respect to conditions typically associated with the σ^B^ response (i.e., NaCl and bile), suggesting that it may work by co-opting and redirecting the pre-existing σ^B^ stress response pathway to cope with additional stressors like antibiotics. How exactly this system operates remains an open question, however, we have ruled out similar models based on research in *S. aureus*, suggesting a novel mode of MazEF action in *L. monocytogenes.*

## 5. Materials and Methods

### 5.1. Bacterial Strains and Growth Conditions

*L. monocytogenes* EGDe wild-type was used in the study (Table 2). Bacterial stock cultures were stored at −80 °C and inoculated on Brain Heart Infusion (BHI; Oxoid CM 1135) agar and grown at 37 °C overnight. An overnight culture was obtained by inoculating one colony in 10 mL BHI broth and incubating aerobically at 37 °C with shaking (250 rpm).

### 5.2. Antibiotic Preparation

Fresh antibiotic solutions were prepared for each experiment: ampicillin (dissolved in sterile MilliQ water; Sigma-Aldrich A9518, St. Louis, MO, USA), gentamicin (dissolved in sterile MilliQ water; Sigma-Aldrich G3632), norfloxacin (dissolved in sterile MilliQ with 1% glacial acetic acid; Fluka N9890), and co-trimoxazole, which is comprised of one part trimethoprim (dissolved in sterile MilliQ with 1% glacial acetic acid; Sigma-Aldrich 92131) and five parts sulphamethoxazole (dissolved in acetone; Fluka S7507).

### 5.3. Constructions of Mutants

In-frame deletion mutants were constructed in the *sigB*, *clpP*, *mazEF* genes using the gene splicing by overlap extension (gene SOEing) method [55]. Primers (Table 3) were constructed using the published sequence of *L. monocytogenes* EGD-e (31). Chromosomal DNA from EGDe was used as the PCR template for the SOEing amplicons, which were subsequently cloned into the pAUL-A vector [54]. Standard protocols were used for plasmid extractions, restriction enzyme digests, and DNA ligations [56]. Plasmids harboring the SOEing fragments were isolated and verified by sequencing. Generation of the deletion mutants was performed as described by Guzman et al. [57], with the exception that electrocompetent cells were prepared as described in Monk et al. [58]. Putative mutants were verified by sequencing at GATC (Koln, Germany). The morphology of each strain was determined to be identical using an Olympus BH2 microscope at 1000X magnification.

### 5.4. Killing Kinetics

Standard killing kinetic experiments were performed as previously described [11], with the exception that the second regrowth and killing step was omitted. In brief, an overnight culture from each strain was diluted 10^6^-fold and grown for 16 h at 37 °C at 250 rpm to obtain an early stationary phase culture. This 16 h culture was diluted to OD_600_ = 0.4 in 2 mL BHI broth and treated with roughly 30 times the Minimum Inhibitory Concentration (MIC) of four bactericidal antibiotics: norfloxacin (100 µg/mL), co-trimoxazole (10 µg/mL), ampicillin (3 µg/mL), and gentamicin (10 µg/mL). Cultures were incubated at 37 °C and 250 rpm during antibiotic treatment. Bacterial counts were determined just before treatment and at subsequent time points by plate counting on BHI agar. The experiment was performed with three biological replicates.

### 5.5. Growth Kinetics

Overnight cultures of each strain were adjusted to OD_600_ = 0.2 and diluted by 10^4^-fold, then the Minimum Inhibitory Concentration (MIC) for each treatment (antibiotics, 42 °C, NaCl, and Bile acid) was identified using a standard two-fold dilution series in 96-well plates using two biological replicates for each strain. Plates were incubated at 37 °C for 24 h in the SpectraMax i3 (Molecular Devices) Multi-Mode microplate reader set to measure OD_600_ every hour. Once the MIC for each treatment was identified, the same procedure above was carried out using two biological replicates, with the exception that the highest concentration started with the MIC and was serially diluted by 10%. Sub-inhibitory concentrations were chosen on the basis of being closest to the MIC, while still allowing for exponential and stationary growth curves of EDGe, Δ*sigB*, and Δ*mazEF*. Antibiotics were prepared as described above, and fresh solutions of NaCl (Merck) and Bile Salts (Cholic acid sodium salt 50% and Deoxycholic acid sodium salt 50%; Fluka, 48305-50G-F) were dissolved in sterile MilliQ water.

### 5.6. Sample Collection and RNA Isolation

To obtain balanced growth, overnight cultures of each strain were diluted 10^9^-fold in 10 mL BHI broth and incubated aerobically at 37 °C with shaking (250 rpm) for 16 h, then subsequently diluted to OD600 0.01 in 50 mL pre-warmed BHI in a 500 mL flask. Upon reaching OD600 0.1, each culture was split into three 100 mL flasks, by taking 7 mL of culture into 23 mL pre-warmed BHI, and further incubated to OD600 0.1. Flasks received either 3 mL MQ water for the no treatment control, or 3 mL MQ water containing NaCl or norfloxacin, yielding a final concentration of 0.5 M or 1 µg/mL, respectively. Each culture was incubated to OD600 0.40, then 5 mL culture was immediately added to 10 mL RNA-protect Bacteria Reagent (Qiagen, 76506, Hilden, Germany) and subsequently treated according to the manufacturer’s instructions. Two independent biological replicates for each strain were carried out.

Cell lysis for downstream RNA purification was carried out by first resuspending each isolated pellet in 500 µL Buffer RLT (Qiagen, 74104) with 5 µL β-meracaptoethanol. Three hundred milligrams of acid washed glass beads were added to the cell suspension, and lysed using the FastPrep FP120 Cell Disruptor. Samples were centrifuged at 4500× *g* for 2 min at 4 °C, then 500 µL of the cell lysis supernatant was mixed with 250 µL 96% ethanol and further processed using the RNeasy mini kit (Qiagen, 74104) according to manufacturer’s protocol “Purification of total RNA from bacteria”, using the additional on column DNase treatment step. RNA quality and quantity were measured using the Agilent 2100 Bioanalyser with a RNA 6000 Nano chip and the DeNovix DS-11+ spectrophotometer, respectively. All RIN values were above 9, with the exception of one replicate of the Δ*clpP* under non-stressed conditions, which had a RIN value of 7.6.

### 5.7. Measuring Relative Gene Expression via Quantitative Reverse Transcription PCR (RT-qPCR)

Prior to cDNA synthesis, 2 µg of RNA from each sample was treated with DNase I (Invitrogen, 18068-015, Carlsbad, CA, USA) and split into reverse transcriptase positive (RT+) and negative (RT−) tubes. cDNA synthesis was carried out for RT+ samples using SuperScript III Reverse Transcriptase (Invitrogen, 18080-044) and random primers (Invitrogen, 48190-011) according to the manufacturer’s protocol for First-Strand cDNA Synthesis. RT− samples were treated identically, with the exception that MQ water was substituted for reverse transcriptase. Prior to qPCR templet cDNA was diluted 4× in MQ water except when investigating 16S, which was diluted 1000×.

RT-qPCR was performed using SYBR Green PCR Master Mix (Applied Biosystems, Foster City, CA, USA) on the Mx3000P (Stratagene, San Diego, CA, USA) using the following program: one cycle at 95 °C for 10 min, followed by 40 cycles at 95 °C for 30 s and 60 °C for 1 min followed by a dissociation curve. Three technical replicates for each RT+ cDNA sample and two technical replicates for RT− cDNA sample were performed for each reaction, along with negative template control using MQ water. Primers (Table 3) were designed using Primer3 [59] and standard curves for each primer set were done using a dilution series of EDGe genomic DNA. 16S gene expression has previously been demonstrated to be stable under stress conditions [60], and was shown in this experiment to be similar between each strain and condition tested using an ANOVA test. 16S gene expression was measured and used to normalize expression levels of each gene measured, which were further normalized to EDGe expression levels using the 2^−ΔΔ*C*t^ method [61]. When comparing the expression of the *mazF* gene alone under different conditions, copy numbers for *mazF* and 16S rRNA gene transcripts were calculated and expressed as target (*mazF*)/housekeeping (16S rRNA gene).

### 5.8. Bioinformatic Analysis of MazF Cut Site Abundance in EDGe Genome

In order to predict the relative abundance of the UACMU motif per gene, as compared to chance, the coding sequences (CDS) of *L. monocytogenes* EDGe (accession No. NC_003210.1) were run through a custom python script based on the formula described in Zhu et al., 2009 [10]:

$$P=\overset{K}{\underset{i=0}{\sum }}p^{i}{\left(1-p\right)}^{L-4-i}\;\frac{\left(L-4\right)!}{i!\left(L-4-i\right)!}$$

Briefly, the probability (*p*) of the UACMU motif occurring in a CDS was calculated using the length (*L*) and base composition, which equals (%U’s)^2^(%A’s)^2^(%C’s) + (%U’s)^2^(%A’s) (%C’s)^2^. Thus, the expected number of motifs in each CDS equals *p*(*L*-4). If *K* is the actual number of motifs found in a CDS, then *P* equals the probability of a CDS containing *K* or fewer motifs. Source code has been uploaded to GitHub [62].

### 5.9. Statistical Analysis

Bacterial counts and OD_600_ measurements from each biological replicate were log_10_ transformed, whereas ΔΔ*C*t values from RT-qPCR were log_2_ transformed, prior to statistical analysis using the macro, Analysis ToolPak, in Microsoft Excel. The F-Test was used to test for equal Variances of the sample populations and Students *t*-test with equal or unequal variance was used when appropriate, with a significance level of *p* < 0.05.

## Figures and Tables

**Table 1:** The top ten genes with the greatest frequency of MazF cleavage sites (*p*), as determined by comparing the actual number of MazF cleavage sites to the predicated number based on gene length and nucleotide content.

Gene Name	Length	UACMU Sites	*p*	Functional Description
*lmo2638*	1887	10	0.9738	NADH dehydrogenase
*secY*	1296	8	0.9436	preprotein translocase subunit SecY
*lmo1310*	1305	8	0.9413	hypothetical protein
*bvrB*	1923	8	0.9303	beta-glucoside-specific phosphotransferase enzyme II ABC component
*lmo1911*	1137	7	0.8988	histidine kinase
*lmo0835*	1005	6	0.8770	peptidoglycan binding protein
*lmo1090*	984	6	0.8698	glycosyltransferase
*lmo1030*	1029	6	0.8600	LacI family transcriptional regulator
*lmo0880*	1389	6	0.8261	wall associated protein precursor
*pgi*	1353	6	0.8259	glucose-6-phosphate isomerase

**Table 2:** Bacterial strains and plasmids used in this study.

Strain or Plasmid	Genotype and Relevant Characteristics	Source or Reference
*E. coli* DH5*α*	Plasmid construction and cloning	Lab stock
*L. monocytogenes* strains		
EGDe	*L. monocytogenes* virulent wild-type BUG1600, Lineage ΙΙ, serotype 1/2a, MLST ST35	O. Dussurget
∆*clpP*	*clpP* deletion mutant derived from *L .monocytogenes* EDGe	This study
∆*mazEF*	*lmo0887 and lmo0888* (*mazEF* antitoxin/toxin) deletion mutant derived from *L. monocytogenes* EDGe	This study
∆*sigB*	*sigB* deletion mutant derived from *L. monocytogenes* EDGe	This study
Plasmids		
pAUL-A	Temperature sensitive origin of replication, *lacZa’* multiple cloning site, erythromycin resistance marker	[54]

**Table 3:** List of primers used in this study.

Primer	Purpose	Sequence (5′- > 3′) ^a,b^
**Mutagenesis**		
ClpP_UpSt_A	A primer: **EcoRΙ**	AAA**GAATTC**CAGTTAATGGGCCAGATT
ClpP_UpSt_B	B primer	ACGAATGGTCAAACTAGG
ClpP_DnSt_C	C primer	CCTAGTTTGACCATTCGTGCACAAAATGCAAAACCTCT
ClpP_DnSt_D	D primer: **BamHΙ**	AAA**GGATCC**CGTGACGGATTATTACCA
ClpP_IC_Fw	Integration and deletion check	TGGCTCTAACGATGATCTTG
ClpP_IC_Rv		TTGATGTTAGTGCACCTGTTG
mazEF_A_Hind	A primer: **Hind ΙΙΙ**	ATGC**AAGCTT**TTAGTAGGCGGGGAACTTGCC
mazEF_B	B primer	TAACACGTGTCACACCCCCAA
mazEF _C	C primer	TTGGGGGTGTGACACGTGTTAGGTTAATGGCTGATGGTGAA
mazEF _D_Bam	D primer: **BamHΙ**	ATGC**GGATCC**TCAGACCCTTTTGCCCTGC
mazEF _IC_Fw	Integration and deletion check	CCTTCCACAGAAATCAAAAC
mazEF _IC_Rv		CCAACCTTTCTCCACTATT
SigB_A_3	A primer: **EcoRΙ**	AAA**GAATTC**AGCTGTAAGTGAAGCCATCAC
SigB_B_3	B primer	CGCCTCTTTATCAGGTTGAGA
SigB_C_3	C primer	TCTCAACCTGATAAAGAGGCGGTGTCTAGAATCCAACGTCAA
SigB_D_3	D primer: **BamHΙ**	AAA**GGATCC**TAATAGCTATCGCAGCACC
SigB_IC_Fw	Integration and deletion check	CGTCAACGCCAAAGTGAA
SigB_IC_Rv		CACCTTTCAAACCATCGCTA
**qPCR**		
mazF_qPCR_Fw	Quantification of *mazF*	ACGGCCTGTTCTCATCATTC
mazF_qPCR_Rv	expression	CGTTGGCAATTTTGCTTTTT
sigB_qPCR_Fw	Quantification of *sigB*	GAAGCAATGGAAATGGGAAA
sigB_qPCR_Rv	expression	CCGTACCACCAACAACATCA
rsbW_qPCR_Fw	Quantification of *rsbW*	ATTACAACTTCCTGCCAAGC
rsbW_qPCR_Rv	expression	AATTGCTTCATAAGAAAATCCTG
opuCA_qPCR_Fw	Quantification of *opuCA*	ACATCGATAAAGGAGAATTTC
opuCA_qPCR_Rv	expression	GCCGGTTAATCATCTTCATTG
2638_qPCR_Fw	Quantification of *lmo2638*	CTGCTGCTACATCTGGTGCT
2638_qPCR_Rv	expression	ACTGGAACCAACCAGGCATA
bvrB_qPCR_Fw	Quantification of *bvrB*	GCAATTGGCGCTAAAACTTC
bvrB_qPCR_Rv	expression	ATTGTAACGATGGCGGTTTC
0880_qPCR_Fw	Quantification of *lmo0880*	ATTCCGAACAAAATGGCAAA
0880_qPCR_Rv	expression	TTTCTGCAACGGAGACATCA

^a^ Bold and underlined indicate restriction sites and the regions binding to the AB fragments in second round PCR, respectively. ^b^ Primers for insertion and integration check were designed in a multi cloning site (MCS) in pAUL-A.
